# Long term risk of recurrence of ptosis repair: implications for surgical counseling and follow-up

**DOI:** 10.3389/fopht.2025.1689010

**Published:** 2026-01-13

**Authors:** Dana Cohen, Maria Laura Passaro, Elena Grachova, Alessia Riccardo, Adriana Iuliano, Vittoria Lanni, Francesco Matarazzo, Antonella D’Aponte, Ciro Costagliola, Diego Strianese

**Affiliations:** 1 Department of Neurosciences, Reproductive Sciences and Dentistry, University of Naples “Federico II”, Naples, Italy; 2 Department of Medicine and Health Sciences “V. Tiberio”, University of Molise, Campobasso, Italy; 3 Taif Eye Center , Amman, Jordan; 4 Department of Physics “Ettore Pancini”, University of Naples “Federico II”, Naples, Italy

**Keywords:** adults, aponeurotic ptosis, congenital ptosis, ptosis, ptosis etiology, ptosis recurrence, ptosis surgery

## Abstract

**Purpose:**

To evaluate recurrence rates after surgical correction of ptosis in adults, with emphasis on differences between aponeurotic and non-aponeurotic etiologies, and to identify predictors of recurrence.

**Methods:**

This retrospective, single-center cohort study included a series of patients undergoing ptosis surgery at single tertiary referral Oculoplastic Unit at University of Naples Federico II (2014–2024). Data collected included demographics, ptosis subtype, surgical technique, preoperative marginal reflex distance (MRD), levator function, systemic comorbidities, and latency to treatment. The primary outcome was recurrence of ptosis. Kaplan–Meier estimates and Cox proportional hazards models were used to evaluate recurrence-free survival and predictors of recurrence. Fisher’s exact test assessed associations with comorbidities.

**Results:**

A total of 122 patients (152 eyes) were included (mean age 59.7 ± 23.8 years; 54.6% male). The median follow-up was 165.1 weeks (IQR 67.0–330.6). Aponeurotic ptosis accounted for 50.7% of eyes, congenital for 30.3%, and myogenic for 18.4%. Levator repair was the most common surgical approach (78.9%). Recurrence occurred in 35 eyes (23.0%) during a median follow-up period of 165.1 weeks (IQR 67.0–330.6), Recurrence was significantly lower in aponeurotic vs non-aponeurotic ptosis (log-rank p = 0.048). In multivariable Cox analysis, non-aponeurotic ptosis was the only independent predictor of recurrence (HR 2.44, 95% CI: 1.13–5.28; p = 0.023). MRD, levator function, latency to treatment, and systemic comorbidities were not meaningfully associated with recurrence.

**Conclusions:**

Ptosis recurrence after surgery was significantly less frequent in aponeurotic ptosis group compared to other forms. Etiology, rather than preoperative assessment measurements and surgical technique, was the primary determinant of long-term outcomes. These findings highlight the importance of etiology-driven surgical counseling and follow-up planning.

## Introduction

Ptosis, or blepharoptosis, refers to the abnormal drooping of the upper eyelid and can significantly impair both function and appearance. In adults, ptosis is not only a cosmetic concern but can cause superior visual field loss, asthenopia, and reduced quality of life. Depending on severity, surgical intervention is the primary management strategy, aiming to restore eyelid position, improve symmetry, and maintain corneal protection (1).

The causes of ptosis are diverse and can be classified as aponeurotic (involutional) or non-aponeurotic. Aponeurotic ptosis, the most common form in older adults, results from attenuation or disinsertion of the levator aponeurosis while the muscle itself remains generally within a normal range function (2). By contrast, congenital ptosis is usually myogenic, caused by maldevelopment of the levator with histological replacement of muscle fibers by fibrous or fatty tissue. Other etiologies include neurogenic ptosis, such as in third nerve palsy or Horner syndrome, and progressive myopathic disorders like oculopharyngeal muscular dystrophy or chronic progressive external ophthalmoplegia (3).

A variety of surgical techniques exist to repair ptosis, with the choice guided by etiology and levator function. Levator advancement is typically employed in aponeurotic ptosis while levator resection is used in congenital cases with fair levator function. In cases of poor levator function, such as severe myogenic ptosis or selected congenital forms, frontalis sling suspension is generally preferred and can be performed using autologous fascia lata or synthetic materials. Frontalis suspension with a silicone rod is also favored by some surgeons, particularly for patients who cannot tolerate longer surgical procedures or who are at higher risk of postoperative corneal exposure, given its reversibility and ease of adjustment. In recent years, frontalis flap suspension has also regained popularity for the management of severe cases with markedly poor levator function (4).

While these procedures are effective, outcomes can vary substantially. Levator-based surgeries for aponeurotic ptosis generally report high success and low recurrence rates, whereas congenital and myogenic ptosis remain challenging, with higher rates of under correction and reoperation (5–7).

This study assesses recurrence rates in a predominantly adult cohort of patients undergoing ptosis repair and investigates the relationships between recurrence, preoperative clinical parameters, and long-term surgical outcomes.

## Materials and methods

### Study design and setting

This retrospective, single-center, registry-based, cohort study was conducted at the Eye Clinic of the University of Naples Federico II, Italy, between December 2024 and February 2025. The study adhered to the tenets of the Declaration of Helsinki. Ethical principles of confidentiality and patient privacy were fully respected throughout the study period.

### Participants

To be included in the study, patients had to have undergone ptosis surgery for the first time, have signed informed consent for the use of their data at the time of surgery, and have complete postoperative follow-up, with all visits recorded in our institutional database. Only patients with successful primary ptosis repair - defined as a satisfactory postoperative outcome characterized by an MRD of 4 mm and symmetrical to the contralateral eyelid - were included in the cohort. Accordingly, patients who required early or late adjustment or reoperation due to failure of the initial procedure were excluded to avoid potential bias. The primary aim of the study was to evaluate the recurrence rate following successful one stage ptosis correction; therefore, an insufficient initial repair was considered an exclusion criterion. Likewise, eyes that had undergone ptosis surgery elsewhere and were reoperated in our institution were not included. All follow-up data were retrieved from our institutional archives, and measurements of eyelid position were obtained in person at postoperative visits. The duration of follow-up used for the analysis was defined as the time from ptosis surgery to the last documented postoperative visit, and this interval is reported in weeks throughout the manuscript. Specifically, patients whose last clinic visit had occurred more than 6 months but no later than 12 months before data collection were contacted by telephone solely to confirm that eyelid position had remained stable since the last documented visit. Patients whose last clinic visit was more than 12 months before data collection and could not be clinically or telephone-verified, as well as those who did not respond or were deceased, were excluded to ensure the accuracy of the last available follow-up time point.

### Pathogenesis grouping and surgical techniques

Aponeurotic, or involutional, ptosis is defined as an acquired form of eyelid drooping caused by attenuation, stretching, or dehiscence of the levator aponeurosis. This form of ptosis occurs most frequently in older adults due to age-related involutional changes, although it may also arise in younger individuals exposed to repeated mechanical stress from long-term contact lens wear, chronic eye rubbing, or prior ocular surgery (8, 9). Clinically, levator excursion is generally preserved and a characteristically elevated or sharply defined eyelid crease reflects retraction of the aponeurotic fibers (8).

Simple congenital ptosis was defined as eyelid drooping present at birth due to maldevelopment of the levator palpebrae superioris muscle (10). Levator function is consistently reduced, and the eyelid often fails to follow the globe appropriately on downgaze, resulting in a high lid position in downward gaze. The eyelid crease is typically absent or poorly formed due to the abnormal insertion of the levator aponeurosis. No systemic neuromuscular disease was present, and the condition was considered an isolated congenital anomaly.

Myogenic ptosis included cases in which the ptosis developed after birth and resulted from a primary myopathic disorder affecting the levator muscle, such as chronic progressive external ophthalmoplegia, mitochondrial myopathies, or muscular dystrophies (McClelland & Cadera, 1981; Petty et al., 1992). These patients generally show markedly reduced levator function along with additional extraocular muscle or facial muscle involvement; as such systemic myopathy was documented as a comorbidity.

Myasthenia gravis–related ptosis is defined as an autoimmune impairment of acetylcholine receptor function which leads to fluctuating weakness of the levator palpebrae superioris, with ptosis that varies in severity over the day and improves with rest (11). Pharmacologic testing with edrophonium or ice-pack testing and electrophysiologic studies provide additional diagnostic confirmation (12). Systemic manifestations were recorded separately as comorbidities.

Neurogenic ptosis included conditions such as third cranial nerve palsy and Horner syndrome. Third nerve palsy typically results in moderate to severe ptosis associated with ophthalmoplegia and, in some cases, pupillary involvement (13). Horner syndrome, by contrast, produces only a mild ptosis due to loss of sympathetic innervation to Müller’s muscle (14). Because the levator palpebrae superioris is not involved in the pathogenesis of Horner-related ptosis, this form was not included in the present cohort.

Mechanical ptosis resulted from physical factors that weighed down the eyelid or restricted its normal excursion, including eyelid tumors, dermatochalasis, scarring, inflammation, or edema. Levator function was generally preserved, but the structural load prevented full eyelid elevation. Because treatment of the underlying mechanical obstruction typically led to improvement in eyelid position, those patients were not included in this cohort.

Traumatic ptosis referred to ptosis caused by direct injury to the eyelid or orbit, which could disrupt the levator muscle, aponeurosis, or the nerves supplying them (15). The severity of ptosis varied widely depending on the extent and location of the damage, and patients having such ptosis were therefore excluded from this study.

Pseudoptosis described conditions in which the eyelid appeared drooped despite normal levator function. This appearance could result from hypotropia, enophthalmos, brow ptosis, redundant upper eyelid skin, or contralateral eyelid retraction, all of which alter the relative eyelid position without representing true ptosis (16–18). This type of ptosis was also excluded from this study.

Three surgeons (DS, VL, and AI) performed all procedures included in this study. Levator repair refers to an anterior-approach levator aponeurosis advancement, performed through an upper-eyelid skin incision with exposure of the levator palpebrae superioris (LPS) complex. The aponeurosis is identified, dissected free from preaponeurotic tissues, and advanced to the superior tarsal border. Fixation is achieved using either nonabsorbable sutures (DS: 5–0 polypropylene) or slowly absorbable sutures (AI and VL: 5–0 polyglactin), according to surgeon preference. When aponeurotic attenuation or significant levator laxity is present, advancement is combined with a limited LPS resection to restore appropriate tension and lid elevation. No posterior Müller’s muscle–conjunctival resections (MMCR) or Whitnall’s sling procedures were performed in this cohort. Although MMCR is an effective technique for select patients, at our center it is reserved for individuals with good levator function and a positive phenylephrine response, and was excluded here to avoid selection bias within an overly specific patient subgroup.

Levator muscle resection, frequently used to correct congenital ptosis by shortening and advancing the levator palpebrae superioris to enhance its eyelid-elevating power, was conducted in this cohort through an anterior eyelid crease incision. Fixation is achieved using either nonabsorbable sutures (DS: 5–0 polypropylene) or slowly absorbable sutures (AI and VL: 5–0 polyglactin), according to surgeon preference.

Frontalis sling procedures were performed using three to five small suprabrow and upper-eyelid margin incisions. A pentagonal sling configuration was created using the closed-sky technique, thereby avoiding a lid crease incision. The suspension material was passed subcutaneously to establish biomechanical coupling between the tarsus and frontalis muscle. Intraoperative tension adjustment was used to optimize eyelid height and contour.

### Data collection

The following variables were collected: age at surgery, gender, eye affected, date of birth, date of surgery, age at ptosis onset, type of ptosis (Simple congenital ptosis, congenital blepharophimosis syndrome, congenital synkinetic ptosis, neurogenic ptosis, myogenic ptosis, aponeurotic ptosis, and mechanical ptosis), surgical treatment type (Levator muscle repair, and Frontal sling operation), preoperative marginal reflex distance (MRD), preoperative levator muscle function, and presence of comorbid conditions including cardiovascular diseases, diabetes mellitus, neoplastic disease, and myopathy.

MRD and levator muscle function were categorized according to clinically relevant thresholds as follows: MRD < 1 mm vs. ≥ 1 mm, and levator function ≤ 3 mm vs. > 3 mm. Levator function ≤ 3 mm was used as a marker of severe levator dysfunction, regardless of etiology.

### Outcome measures

Recurrence was defined as a decrease of at least 1 mm in MRD relative to the postoperative measurement obtained after an initially successful ptosis repair. By using this criterion, recurrence was identified regardless of whether the patient ultimately underwent a secondary corrective procedure. Consequently, all patients who required reoperation for recurrent eyelid drooping were, by definition, included in the recurrence group. Importantly, the inclusion of patients who demonstrated a progressive decline in eyelid height but did not undergo reintervention minimizes the risk of underestimating recurrence, particularly in individuals who may tolerate a mild (1–2 mm) degree of ptosis and are therefore less inclined to pursue additional surgery—an effect especially relevant among older adults. Secondary outcome measures included the comparison of recurrence-free survival across ptosis subtypes using Kaplan–Meier estimates, and the identification of independent predictors of recurrence, including MRD, levator muscle function, and latency to treatment (defined as the time between ptosis onset and surgery). Additionally, the association between systemic comorbidities and the risk of recurrence was examined.

### Statistical analysis

Data were analyzed using R software version 4.2.2 (R Project for Statistical Computing, Vienna, Austria). Categorical variables were summarized as frequencies and percentages, while continuous variables were described using means and standard deviations.

For the purposes of survival analysis, ptosis subtypes were dichotomized into two groups: aponeurotic ptosis and all other subtypes. The primary outcome—ptosis recurrence—was analyzed using Kaplan–Meier survival estimates and compared between groups with the log-rank test.

A Cox proportional hazards model was subsequently constructed to assess the association between ptosis type (aponeurotic vs other), MRD, levator muscle function, and latency to treatment (defined as the time in years between ptosis onset and surgery). Hazard ratios (HRs) with corresponding 95% confidence intervals (CIs) were calculated, and the proportional hazards assumption was verified.

Additionally, the association between systemic comorbidities and recurrence was explored using Fisher’s exact test. A p-value < 0.05 was considered statistically significant.

## Results

### Demographic and clinical features

A total of 161 eyes of patients who had undergone surgical correction of ptosis were initially identified through a retrospective review of surgical and clinical records. Of these, 9 eyes belonged to patients that were excluded due to being unreachable or deceased at the time of follow-up. The final study population included 122 patients, accounting for 152 eyes.

The mean age at the time of surgery was 59.7 years (SD 23.8), and male patients represented 54.6% of the cohort population. The mean age at ptosis onset was 42.2 years (SD 28.6). The median follow-up was 165.1 weeks (IQR 67.0–330.6). The most commonly performed surgical procedure was levator palpebrae muscle repair (78.9%), followed by frontal sling (21.1%). Preoperative assessments demonstrated a mean marginal reflex distance (MRD) of 0.93 mm (SD 1.01) and a mean levator muscle function of 7.98 mm (SD 4.67).

Systemic comorbidities were frequently observed: cardiovascular diseases were present in 34.2% of patients, type 2 diabetes mellitus in 4.6%, neoplastic diseases in 7.9%, and myopathy in 5.9%.

The cohort included the following distribution of ptosis subtypes: aponeurotic ptosis in 77 eyes (50.7%), simple congenital ptosis in 46 eyes (30.3%), myogenic ptosis in 28 eyes (18.4%), neurogenic ptosis in 1 eye (0.7%). No cases of congenital blepharophimosis syndrome, congenital synkinetic ptosis, or mechanical ptosis were present.

The median follow-up duration was 165.1 weeks (IQR: 67.0–330.6 weeks), allowing for adequate long-term evaluation of surgical outcomes. A comprehensive summary of baseline characteristics is presented in **Table 1**.

**Table 1:** Demographic and clinical characteristics of the study cohort.

Characteristic	Overall, N = 152^1^	Simple congenital ptosis, N = 46^1^	Neurogenic ptosis, N = 1^1^	Myogenic ptosis, N = 28^1^	Aponeurotic ptosis, N = 77^1^
Age at surgery	60 (24)	37 (22)	60 (NA)	62 (19)	73 (15)
Gender					
f	69 (45%)	19 (41%)	1 (100%)	14 (50%)	35 (45%)
m	83 (55%)	27 (59%)	0 (0%)	14 (50%)	42 (55%)
Onset age	42 (29)	5 (11)	60 (NA)	46 (22)	63 (11)
Follow-up (weeks)	165.07 [67.00, 330.57]	129.79 [66.57, 313.57]	91.29 [91.29, 91.29]	93.71 [50.68, 280.21]	265.57 [88.57, 363.43]
Treat					
Levator repair	120 (79%)	33 (72%)	1 (100%)	17 (61%)	69 (90%)
Frontal sling	32 (21%)	13 (28%)	0 (0%)	11 (39%)	8 (10%)
MRD	0.93 (1.01)	1.11 (1.08)	0.00 (NA)	0.82 (0.94)	0.87 (0.99)
Levator function	8.0 (4.7)	6.6 (4.5)	10.0 (NA)	5.4 (4.4)	9.7 (4.3)
Cardiovascular diseases					
Absent	100 (66%)	40 (87%)	1 (100%)	21 (75%)	38 (49%)
Present	52 (34%)	6 (13%)	0 (0%)	7 (25%)	39 (51%)
Diabetes mellitus					
Absent	145 (95%)	45 (98%)	1 (100%)	28 (100%)	71 (92%)
Present	7 (4.6%)	1 (2.2%)	0 (0%)	0 (0%)	6 (7.8%)
Neoplastic disease					
Absent	140 (92%)	44 (96%)	1 (100%)	23 (82%)	72 (94%)
Present	12 (7.9%)	2 (4.3%)	0 (0%)	5 (18%)	5 (6.5%)
Myopathy					
Absent	143 (94%)	46 (100%)	1 (100%)	19 (68%)	77 (100%)
Present	9 (5.9%)	0 (0%)	0 (0%)	9 (32%)	0 (0%)

^1^Mean (SD); median [IQR]; n (%).

Continuous variables are reported as means and standard deviations or medians and interquartile ranges, as appropriate. Categorical variables are reported as absolute counts and percentages. Data are presented overall and stratified by ptosis subtype.

### Recurrence and survival analysis

Of the 152 eyes included, a total of 35 cases (23.0%) experienced ptosis recurrence during the follow-up period. Among these, 15 recurrences (42.85%) occurred in the aponeurotic ptosis group (n = 77), whereas 20 recurrences (57.14%) were observed in the group with other types of ptosis (n = 75).

**Figure 1:**
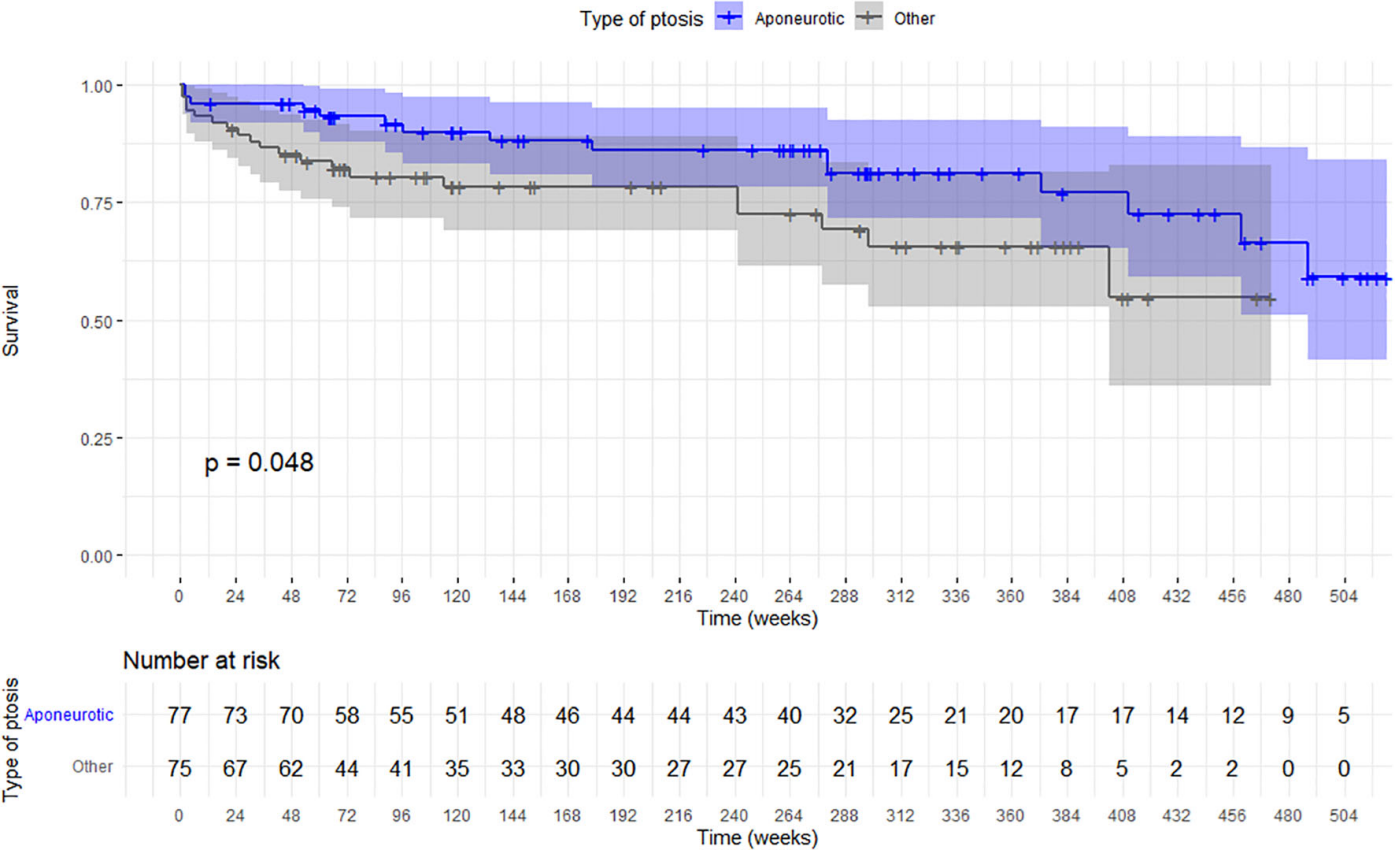
Kaplan–Meier curves of recurrence-free survival after ptosis surgery stratified by etiology. The y-axis represents the probability of recurrence-free survival, and the x-axis represents follow-up time in weeks. Patients with aponeurotic ptosis (blue line) showed significantly higher recurrence-free survival compared with those with other etiologies (gray line). Shaded areas represent 95% confidence intervals. The table below each curve reports the number of eyes at risk at different time points throughout follow-up (in weeks).

Kaplan–Meier survival curves were generated to estimate recurrence-free survival stratified by ptosis type (**Figure 1**). Although the median survival time was not reached in either group, the curves demonstrated a trend toward improved long-term outcomes in patients with aponeurotic ptosis. The log-rank test showed a statistically significant difference between the two groups (p = 0.048), indicating a lower cumulative incidence of recurrence among patients with aponeurotic ptosis.

A Cox proportional hazards model was constructed including ptosis type (aponeurotic vs other), MRD, preoperative levator muscle function, and latency to treatment. Given the strong correlation between ptosis subtype and age (**Supplementary Figure 1**), age was not included as a covariate in the model to avoid collinearity. Indeed, the mean age at onset was substantially higher in the aponeurotic group (mean 62.8 years, SD 11.1) compared to the group with other ptosis types (mean 21.3 years, SD 25.7).

In the multivariable model, ptosis type remained the only independent predictor of recurrence. Patients with non-aponeurotic ptosis had a significantly increased risk of recurrence compared to those with aponeurotic ptosis (HR = 2.44, 95% CI: 1.13–5.28; p = 0.023). As expected, mean levator function was higher in aponeurotic cases, consistent with the preserved muscular anatomy of this group; however, preoperative levator function ≤ 3 mm was not significantly associated with recurrence (HR = 0.63, 95% CI: 0.26–1.53; p = 0.31). Similarly, patients with MRD < 1 mm showed no significant difference in recurrence risk compared to those with MRD ≥ 1 mm (HR = 1.24, 95% CI: 0.62–2.48; p = 0.54). Latency to treatment, expressed as the number of years between onset and surgery, was also not associated with recurrence (HR = 0.99 per year, 95% CI: 0.97–1.01; p = 0.48). Results are presented in **Table 2**.

**Table 2:** Multivariable Cox proportional hazards model for the risk of ptosis recurrence.

Characteristic	HR^1^	95% CI^1^	p-value
Ptosis type (Other vs Aponeurotic)			
Aponeurotic	—	—	
Other	2.44	1.13, 5.28	0.023
Levator function (≤ 3 mm vs > 3 mm)			
Levator Function > 3 mm	—	—	
Levator Function ≤ 3 mm	0.63	0.26, 1.52	0.3
MRD (< 1 mm vs ≥ 1 mm)			
MRD ≥ 1 mm	—	—	
MRD < 1 mm	1.24	0.62, 2.48	0.5
Latency to treatment (years)	0.99	0.97, 1.01	0.5

^1^HR, Hazard Ratio; CI, Confidence Interval.

Hazard ratios (HRs) and 95% confidence intervals (CIs) are reported for each variable included in the model.

### Comorbidities and recurrence

**Figure 2:**
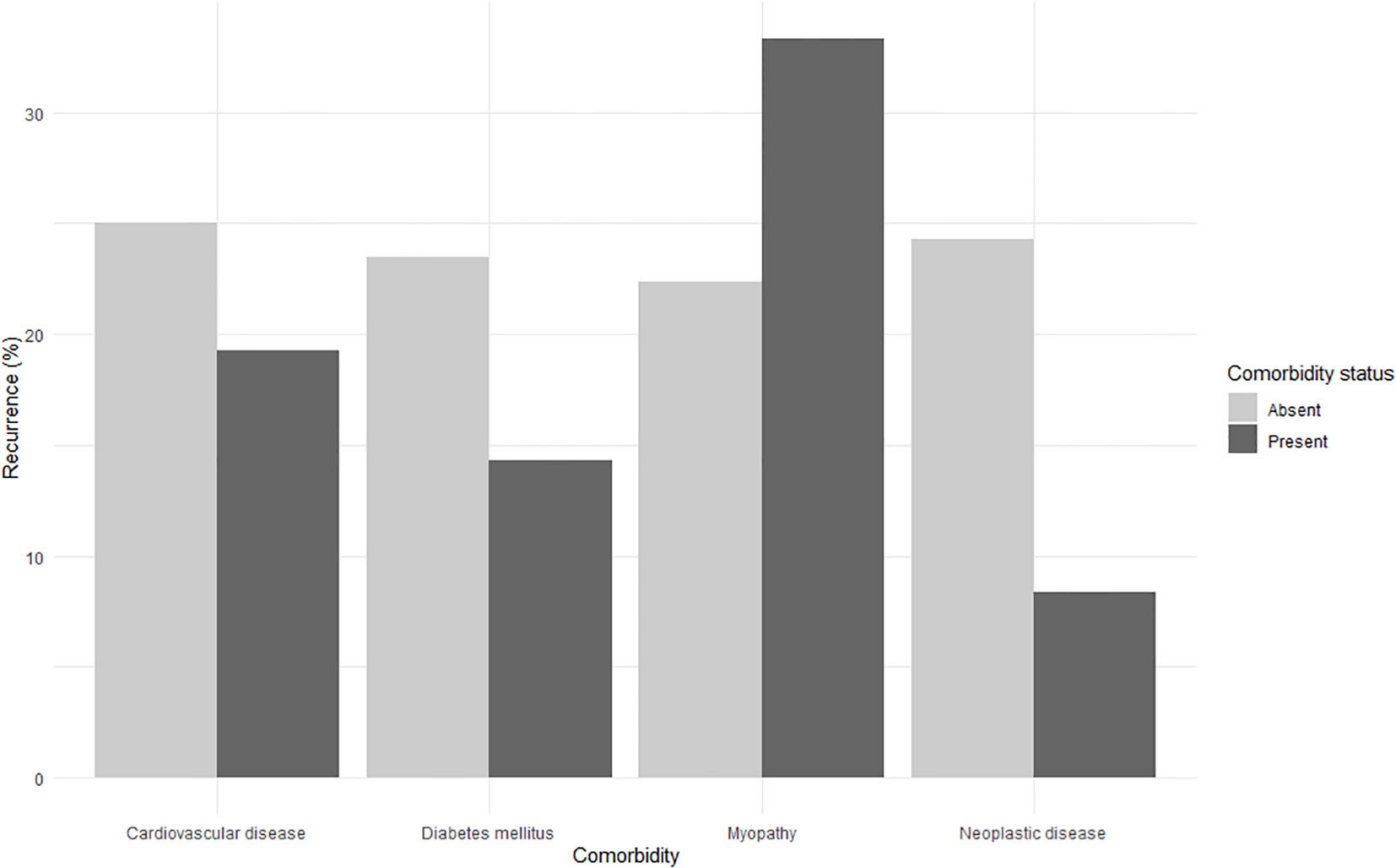
Recurrence rates by comorbidity status. Recurrence rates (%) after ptosis surgery according to the presence or absence of cardiovascular disease, diabetes mellitus, myopathy, and neoplastic disease. The y-axis represents the percentage of eyes with recurrence, and the x-axis lists the comorbidities analyzed.

The relationship between systemic comorbidities and ptosis recurrence was explored using Fisher’s exact test. None of the conditions analyzed showed a statistically significant association with recurrence. Cardiovascular disease was present in 34.2% of patients, with a recurrence rate of 19.2% in affected individuals versus 25.0% in those without (p = 0.54). Among patients with diabetes mellitus (4.6%), recurrence occurred in 14.3% compared to 23.5% in non-diabetic patients (p = 1.00). Similarly, patients with neoplastic disease (7.9%) had a recurrence rate of 8.3%, compared to 24.3% in those without (p = 0.30). Myopathy, present in 5.9% of cases, was associated with a recurrence rate of 33.3%, versus 22.4% in those without (p = 0.43). A descriptive visualization of recurrence rates stratified by comorbidity status is shown in **Figure 2**.

## Discussion

In our study, 152 eyes of adult patients were analyzed, divided into two nearly equal groups: 77 eyes with aponeurotic ptosis and 75 with other etiologies of ptosis. The demographic profile of the groups differed significantly. Aponeurotic ptosis was primarily seen in older adults (mean age 73 years), reflecting the age-related nature of this condition, while other etiologies, such as congenital or myogenic ptosis, appeared at a younger age (mean age 46.6 years).

Aponeurotic ptosis encompasses three related structural failures of the levator aponeurosis. The first mechanism is aponeurotic disinsertion, which refers to complete or near-complete separation of the aponeurosis from the anterior tarsal plate. Clinically, patients often demonstrate a high eyelid crease and good levator function. Surgical correction implies levator advancement with direct reattachment of the aponeurosis to the superior third of the tarsus. The second mechanism is aponeurotic dehiscence, typically representing partial or zonal splitting of aponeurotic fibers, often seen in involutional or post-surgical ptosis. Levator function remains preserved, but transmission of force is weakened. Surgical correction includes advancement or plication of the levator aponeurosis, with reinforcement of the attenuated fibers and restoration of tension. The third mechanism is aponeurotic degeneration (attenuation or thinning), which may result from chronic stretching or age-related involution and can cause diffuse thinning of the aponeurosis without a discrete tear. Surgical correction requires levator resection or advancement to compensate for the reduced tensile strength of the attenuated aponeurosis. Resection provides both tightening and functional reinforcement of the aponeurotic–muscular complex. Across these three aponeurotic mechanisms, the underlying levator muscle remains structurally preserved, which explains the excellent outcomes and low recurrence rates we observed in the aponeurotic group.

Importantly, in our cohort, stratification of patients into these three aponeurotic subtypes did not demonstrate any meaningful differences in recurrence rates or surgical outcomes. This supports the view that once the aponeurotic–tarsal interface is surgically restored—whether through reattachment, tightening, or resection—the prognosis is uniformly favorable across all forms of aponeurotic failure. This further reinforces our conclusion that aponeurotic ptosis uniformly carries a lower recurrence risk than non-aponeurotic etiologies, independent of its specific anatomical mechanism.

Frontalis sling procedure is not the standard first-line treatment for aponeurotic ptosis. However, in our series, approximately 10% of patients with aponeurotic ptosis underwent frontalis sling based on specific clinical considerations. These were predominantly elderly patients with poor levator function, multiple comorbidities, and limited tolerance for prolonged procedures under local anesthesia. In such cases, the frontalis sling was selected as a quicker, minimally invasive, and reversible option, typically performed bilaterally in less than 10 minutes. This technique offers the additional advantage of being rapidly reversible—simply by cutting the silicone rod—which is particularly valuable in elderly patients at high risk of corneal exposure. The ability to promptly reverse a potentially threatening eyelid position provides an important safety margin in this population.

The overall recurrence rate in our cohort was 23%, consistent with prior reports across mixed ptosis populations (6). Importantly, we found a statistically significant lower recurrence rate in the aponeurotic group compared with the non-aponeurotic group, independent of the surgical technique chosen. Our multivariable analysis further supported these findings. When adjusting for baseline characteristics such as MRD, preoperative levator muscle function, and latency to treatment, ptosis type emerged as the only independent predictor of recurrence. Patients with non-aponeurotic ptosis demonstrated a distinctly higher likelihood of recurrence compared with those with aponeurotic ptosis, reinforcing the conclusion that etiology, rather than anatomical measurements or timing of intervention, primarily dictates surgical prognosis.

Lagophthalmos following ptosis repair is a well-recognized complication, especially among vulnerable patients, and may lead to corneal problems in the presence of extraocular movement disorders, weak orbicularis function, poor Bell’s phenomenon, or incomplete eyelid closure. In patients with poor Bell’s phenomenon, postoperative lagophthalmos can result in significant exposure-related complications, including dryness and ulceration. Therefore, the decision to use frontalis sling in these aponeurotic cases was made after careful assessment of these risk factors and thorough discussion with the patients.

Although Müller’s muscle–conjunctival resection is a well-established and effective technique for ptosis correction, patients who underwent this procedure were not included in our cohort. At our center, this approach is typically reserved for individuals with good levator function and a positive phenylephrine test. To avoid selection bias stemming from these highly specific indications, we excluded patients treated with Müller’s muscle–conjunctival resection. We plan to further evaluate and directly compare the outcomes of anterior and posterior approaches in a future head-to-head study. To our knowledge, no previous adult series has explicitly reported this difference regardless of procedure. This suggests that the underlying cause of ptosis exerts more influence on surgical prognosis than the type of repair employed, supporting the view that etiology is the dominant factor in recurrence risk.

In interpreting recurrence rates, it is important to note that our definition was more stringent than in many previous studies, which often considered only reoperation. By defining recurrence as any decline ≥1 mm from the postoperative MRD, we also captured patients with progressive drooping who did not undergo further surgery, thereby reducing underestimation in older or less intervention-seeking individuals. In term of surgical management of recurrence, most cases were treated with a repeat of the initial procedure. For eyes originally treated with levator muscle resection that later showed a further decrease of levator function to less than 3 mm, frontalis sling suspension or frontalis flap suspension was offered and discussed instead of a repeat levator resection. Only two patients in this cohort needed a third revision of the levator advancement procedure.

Previous literature on adult ptosis has largely evaluated outcomes by surgical method. For example, large datasets of adult levator repairs have confirmed low revision rates, typically around 7–10% (19). By contrast, sling procedures in adults have shown markedly higher complication rates, including extrusion up to 14% and reoperation approaching 19% (8,9). Our study adds nuance to this body of evidence by demonstrating that when controlling for etiology, the difference in recurrence is more closely linked to why the ptosis developed rather than how it was repaired. This represents a novel contribution to the literature on adult ptosis outcomes.

In aponeurotic ptosis, the levator muscle is preserved, and recurrence is rare once the aponeurosis is securely reattached (1,3). In contrast, congenital and myogenic ptosis involve structural deficiencies of the muscle itself, with fibrofatty replacement and poor contractile capacity (2,4). Even technically successful surgeries—whether levator resections or slings—cannot fully overcome this biological limitation, which explains the higher recurrence and reoperation rates reported in these groups (5,6,7,9).

Poor levator function was not uniformly due to a primary myopathic process. Only a subset of eyes with levator excursion ≤ 3 mm had true myogenic ptosis, whereas many low measurements occurred in simple congenital ptosis or in long-standing non-myopathic ptosis with secondary disuse. In our analysis, levator function ≤ 3 mm therefore reflects severe functional impairment from heterogeneous causes, rather than defining a specific etiological category. All patients with levator function ≤ 3 mm in this cohort underwent frontalis sling suspension, which bypasses the levator mechanism by coupling eyelid elevation to frontalis action. As postoperative eyelid height was primarily supported by the frontalis muscle rather than levator recovery, recurrence was not directly driven by the preoperative levator measurement in these cases. This, together with the relatively small proportion of eyes with documented systemic myopathy (5.9%), likely reduced the power of the model to detect an independent association between levator function, myopathic disease, and recurrence.

Taken together, our findings consistently showed that the type of ptosis - aponeurotic versus non-aponeurotic - was the dominant predictor of recurrence. This underscores that levator function alone is an imperfect proxy for the underlying biological process driving recurrence risk and that etiology provides a more clinically meaningful framework for prognostication. Our data therefore emphasize that in adult ptosis, surgical prognosis is determined by etiology more than by surgical technique. Clinically, this means patients with aponeurotic ptosis can be counseled about the durability of a single operation, while patients with congenital or myogenic etiologies should be informed of the higher likelihood of recurrence and the need for further interventions. By framing outcomes in terms of etiology, surgeons can provide clearer expectations and tailor follow-up more effectively.

One limitation of this study is that several commonly employed ptosis repair techniques were not represented, as they were not utilized by the surgeons participating in the investigation. In general, the choice of procedure was strongly influenced by ptosis etiology and preoperative levator function, as well as by individual surgeon preference. Levator repair was performed almost exclusively in aponeurotic ptosis, whereas frontalis sling procedures were largely reserved for congenital and myogenic cases. As a results, the effects of surgical technique and underlying etiology are difficult to separate, and the non-randomized allocation of procedures may have contributed to differences in recurrence rates. This potential selection bias should be taken into account when interpreting the findings.

In summary, this study demonstrates that aponeurotic ptosis in adults carries a lower recurrence risk compared to other etiologies, irrespective of pre-surgical measurements and the surgical method employed. This insight reinforces the importance of etiology-driven surgical planning, adds a novel observation to the adult ptosis literature, and underlines the need to stratify future outcome studies by cause of ptosis rather than procedure alone.

## Data Availability

The data analyzed in this study is subject to the following licenses/restrictions: The datasets generated and analyzed during the current study are not publicly available due to patient confidentiality and institutional regulations, but de-identified data may be made available from the corresponding author on reasonable request. Requests to access these datasets should be directed to diego.strianese@unina.it.

## Supplementary material

Supplementary Figure 1 Distribution of age at onset by ptosis type. Boxplot showing the age at onset in patients with aponeurotic ptosis compared with those with other etiologies (congenital, myogenic, neurogenic).
