# Supplementary figures and images for: Long term risk of recurrence of ptosis repair: implications for surgical counseling and follow-up
Source: Front Ophthalmol (Lausanne). 2026 Jan 13;5:1689010. doi: 10.3389/fopht.2025.1689010 (PMC12835895; doi:10.3389/fopht.2025.1689010)

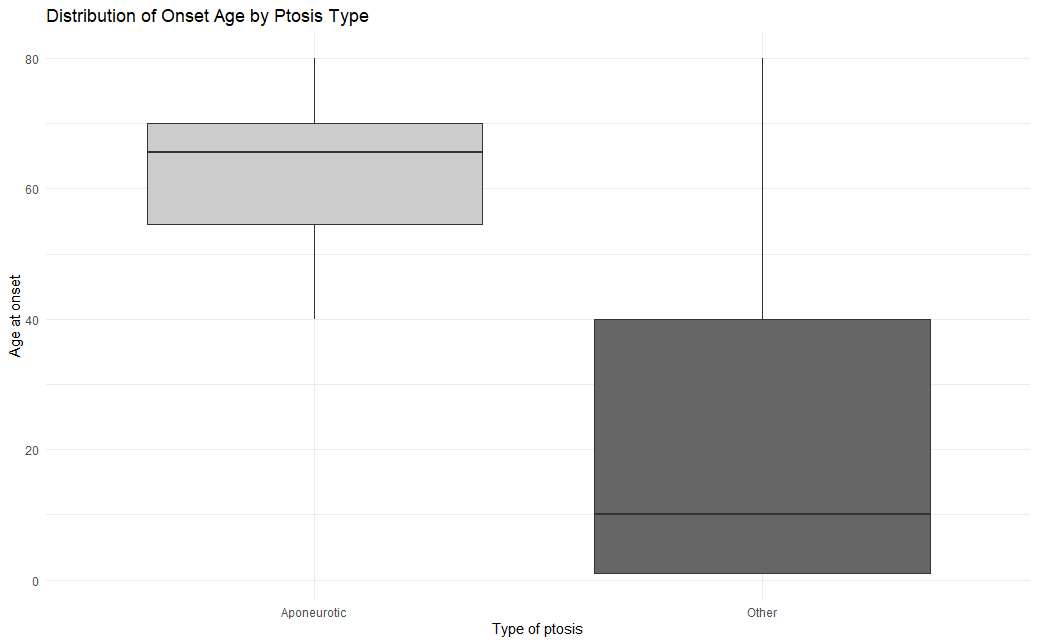

Supplement: Supplementary Figure 1 — Distribution of age at onset by ptosis type. Boxplot showing the age at onset in patients with aponeurotic ptosis compared with those with other etiologies (congenital, myogenic, neurogenic). [file Image1.tiff]
